# Supplementary material for: Synergistic Interferon-Alpha-Based Combinations for Treatment of SARS-CoV-2 and Other Viral Infections
Source: Viruses. 2021 Dec 11;13(12):2489. doi: 10.3390/v13122489 (PMC8705725; doi:10.3390/v13122489)
Supplement: Supplementary file 1 [file viruses-13-02489-s001.zip › viruses-1483518-supplementary.pdf]

Supplementary file

Table S1. Compounds, their suppliers, and catalogue numbers.

Name	Cat #	Company
Recombinant human IFNa1b	11343594	ImmunoTools
Recombinant human IFNa2a	11343504	ImmunoTools
Recombinant human IFNa2b	11343514	ImmunoTools
Recombinant human IFNb1a	11343520	ImmunoTools
Recombinant human IFNb1b	11343542	ImmunoTools
Recombinant human IFNg	11343534	ImmunoTools
Recombinant human IFNw1	11344784	ImmunoTools
Recombinant human IL28A	11340280	ImmunoTools
Recombinant human IL-29	11340290	ImmunoTools
Recombinant mouse IFNaa	10150-IF-050	R&D Systems
Camostat mesylate	16018	Cayman Chemicals
Remdesivir	30354	Cayman Chemicals
Lamivudine	S1706	Selleckchem
Cycloheximide	C7698-1g	SigmaAldrich
Pimodivir	HY-12353A/CS	MedChemExpress
EIDD-2801	HY-135853	MedChemExpress
Ribavirin	0219606650	MP Biomedicals
NITD008	SML2409	SigmaAldrich
Sofosbuvir	S2794	Selleckchem
Telaprevir	S1538	Selleckchem

Table S2. Statistical analysis of data presented in Fig. 1 using pairwise Wilcoxon Rank Sum Test with continuity correction.

**Calu-3**	IFNa1b	IFNa2a	IFNa2b	IFNb1a	IFNb1b	IFNg	IFNw1	IL28a	IL29	IP10
IFNa2a	0,08	-	-	-	-	-	-	-	-	-
IFNa2b	0,70	0,38	-	-	-	-	-	-	-	-
IFNb1a	0,10	0,66	0,40	-	-	-	-	-	-	-
IFNb1b	1,00	0,18	0,40	0,10	-	-	-	-	-	-
IFNg	1,00	0,18	1,00	0,40	1,00	-	-	-	-	-
IFNw	0,40	0,18	0,83	0,10	1,00	0,70	-	-	-	-
IL28a	0,51	1,00	0,40	0,83	0,40	0,40	0,70	-	-	-
IL29	0,27	0,38	0,70	0,10	0,40	1,00	1,00	0,70	-	-
IP10	0,20	0,64	0,40	0,83	0,40	0,27	0,70	1,00	0,40	-
No_drug	0,51	0,38	1,00	0,12	0,40	1,00	1,00	0,51	1,00	0,40

**Vero-E6**	IFNa1b	IFNa2a	IFNa2b	IFNb1a	IFNb1b	IFNg	IFNw1	IL28a	IL29	IP10
IFNA2a	0,10	-	-	-	-	-	-	-	-	-
IFNA2b	0,10	1,00	-	-	-	-	-	-	-	-
IFNB1a	0,08	0,38	0,38	-	-	-	-	-	-	-
IFNB1b	0,20	0,10	0,10	0,08	-	-	-	-	-	-
IFNg	0,12	0,27	0,40	0,18	0,83	-	-	-	-	-
IFNW1	0,70	0,20	0,27	0,18	0,83	1,00	-	-	-	-
IL28a	0,20	1,00	1,00	1,00	0,70	1,00	0,51	-	-	-

IL29	0,08	0,26	0,38	0,18	0,08	0,50	0,38	1,00	-	-
IP10	0,10	0,40	0,70	0,38	0,10	0,70	0,40	1,00	1,00	-
No_drug	0,10	1,00	1,00	0,66	0,27	0,51	0,70	1,00	1,00	1,00

Table S3. Statistical analysis of data presented in Fig. 2 using pairwise Wilcoxon Rank Sum Test.

**Mock, p1**	IFNa1b	IFNa2a	IFNb1a	IFNg	no
IFNa2a	0,0022	-	-	-	-
IFNb1a	0,9372	0,0022	-	-	-
IFNg	0,0022	0,9372	0,0022	-	-
no	0,0022	0,9372	0,0022	0,8182	-
IFNw1	0,3095	0,0087	0,4848	0,0022	0,0087

**Virus, p1**	IFNa1b	IFNa2a	IFNb1a	IFNg	no
IFNa2a	0,0022	-	-	-	-
IFNb1a	0,1797	0,0022	-	-	-
IFNg	0,6991	0,0087	0,0022	-	-
no	0,0022	0,6991	0,0022	0,0043	-
IFNw1	0,6991	0,0022	0,1797	0,4848	0,0022

**Mock, p2**	IFNa1b	IFNa2a	IFNb1a	IFNg	no
IFNa2a	0,065	-	-	-	-
IFNb1a	0,699	0,065	-	-	-
IFNg	0,31	0,485	0,24	-	-
no	0,093	0,937	0,093	0,394	-
IFNw1	0,589	0,132	0,699	0,589	0,18

**Virus, p2**	IFNa1b	IFNa2a	IFNb1a	IFNg	no
IFNa2a	0,3095	-	-	-	-
IFNb1a	0,4848	0,026	-	-	-
IFNg	0,5887	0,4848	0,0649	-	-
No	0,0649	0,3939	0,0087	0,1797	-
IFNw1	0,5887	0,0087	1	0,026	0,0087

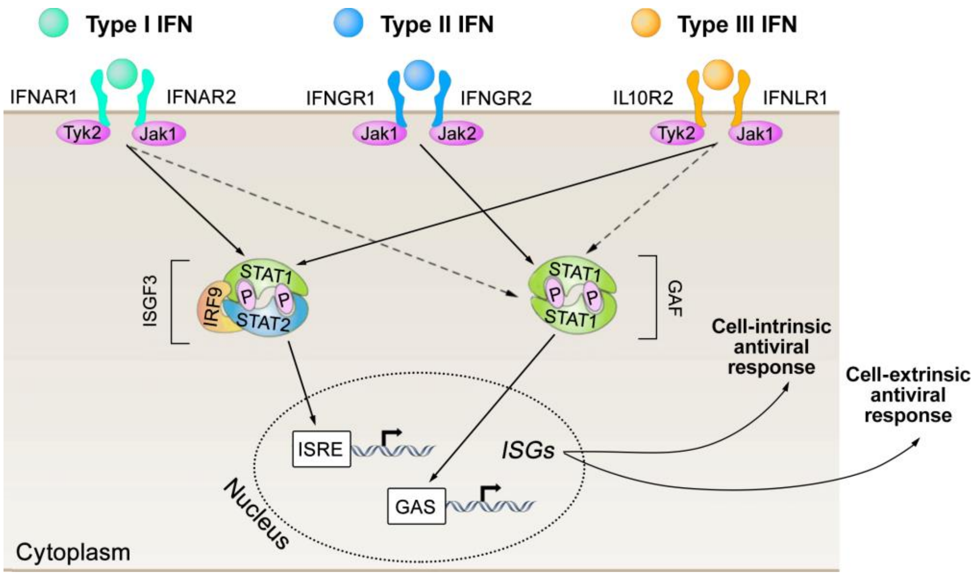

Figure S1. Three types of IFNs and their targets (adapted from [10]).

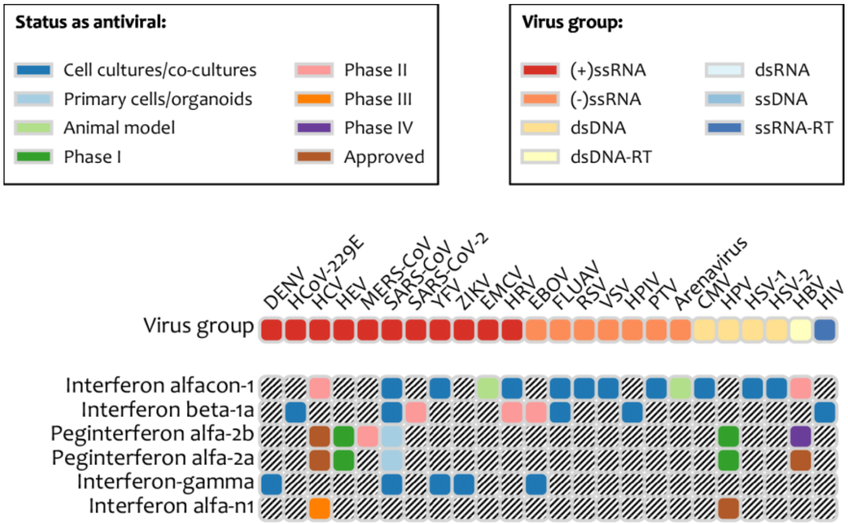

Figure S2. Developmental statuses of several natural and recombinant human IFNs against a range of pathogenic human viruses. Data was retrieved from our database of broad-spectrum antivirals (http://drugvirus.info/).

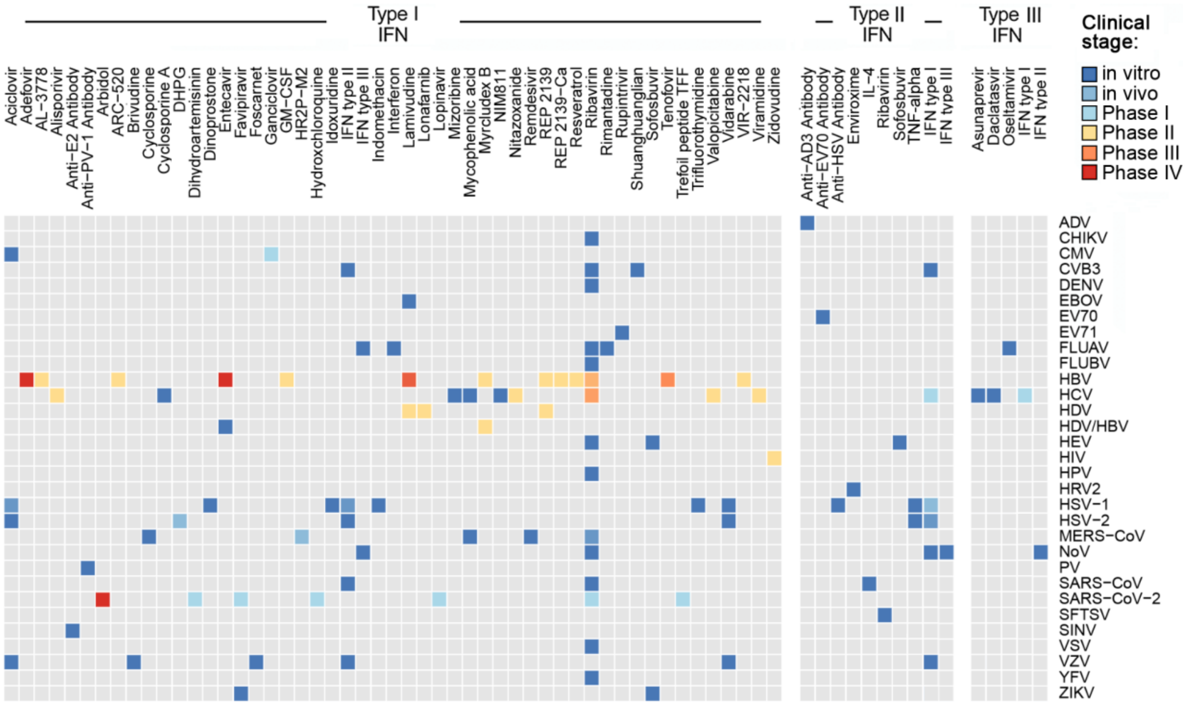

Figure S3. Examples of IFN-based combinations and their developmental statuses. Data was retrieved from our antiviral drug combinations database (https://antiviralcombi.info).

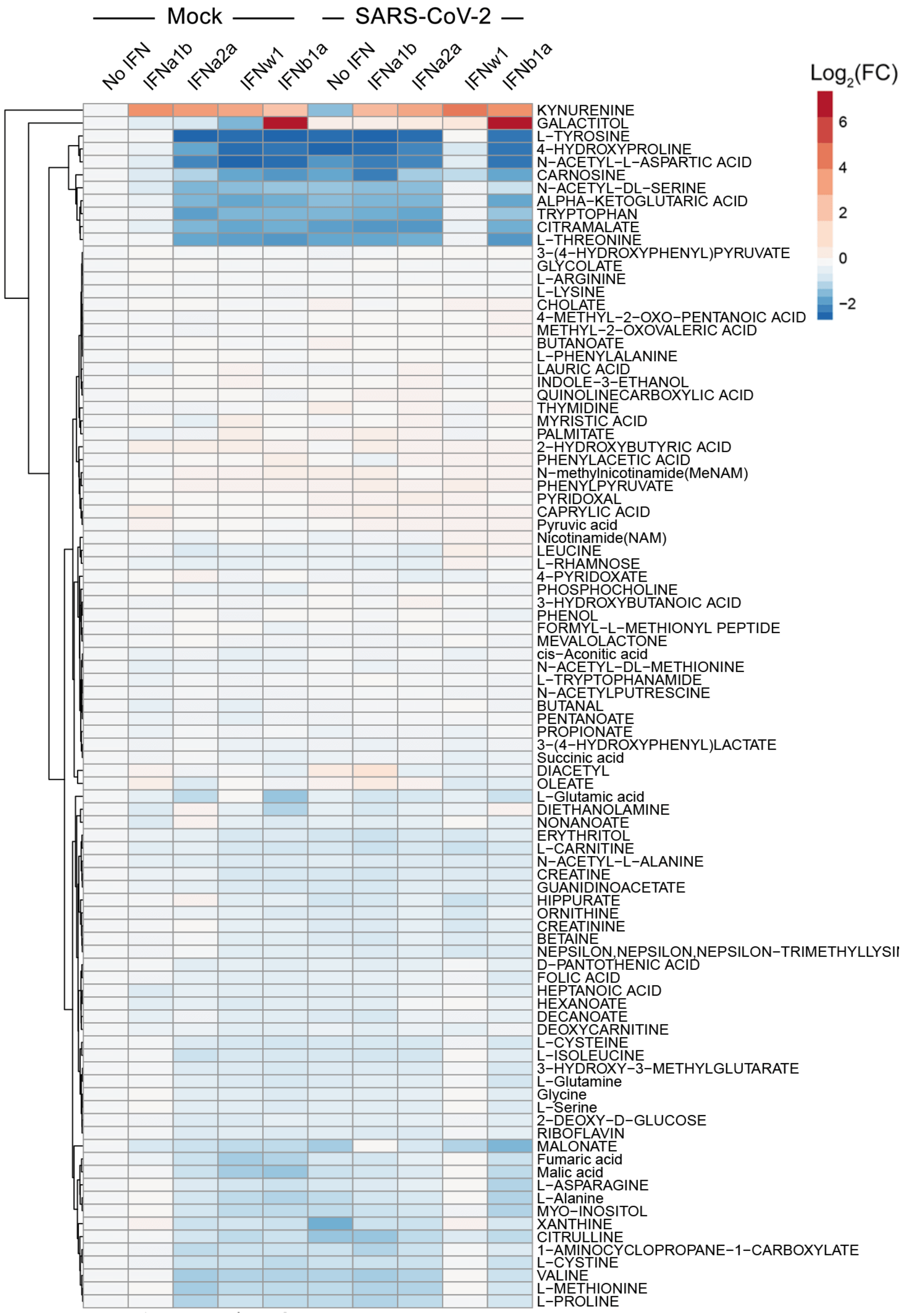

Figure S4. Metabolomic profiles of mock- and SARS-CoV-2-infected Calu-3 cells non-treated or treated with type I IFNs.

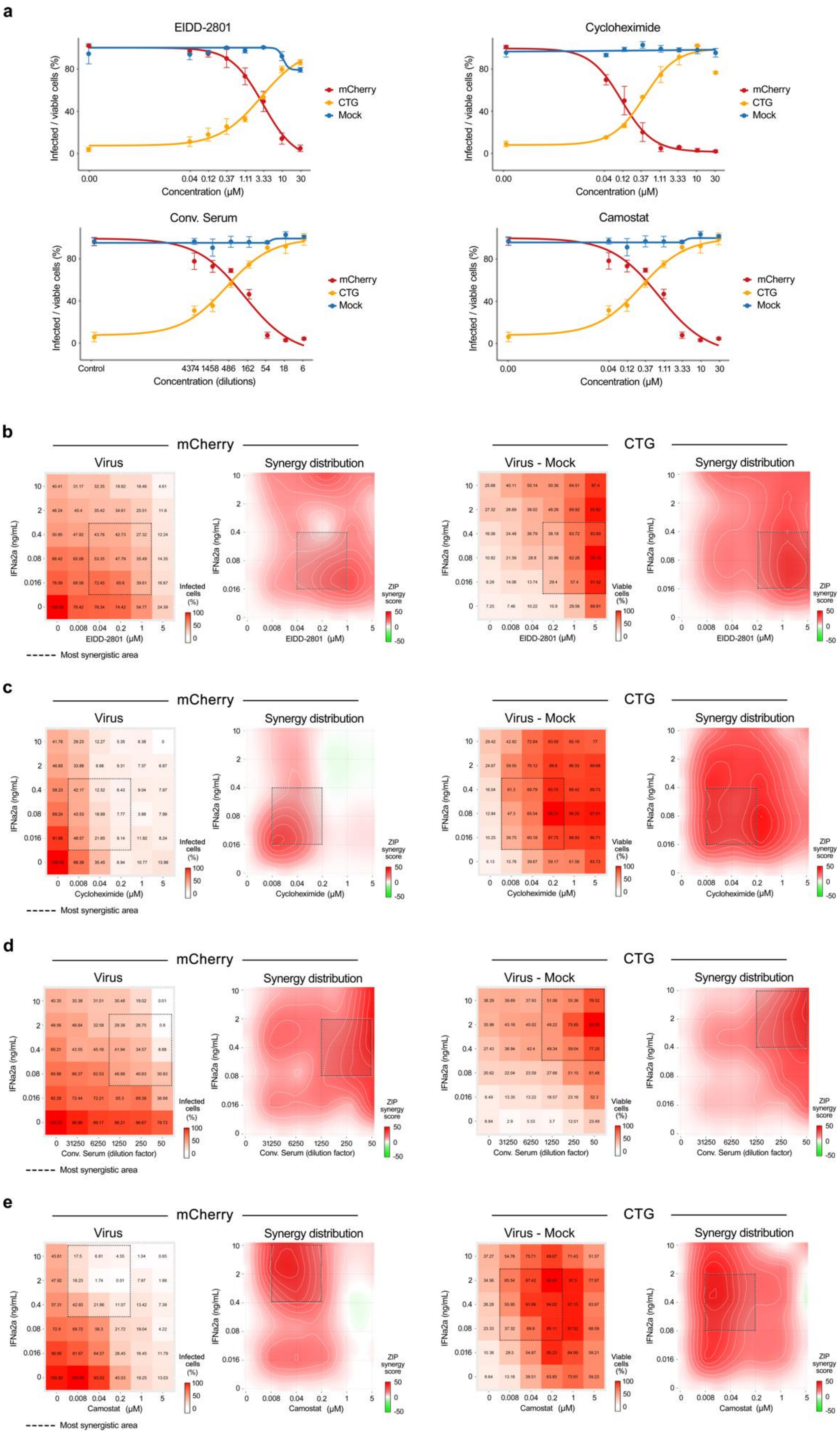
a
EIDD-2801
Cycloheximide
Conv. Serum
Camostat
Infected / viable cells (%)
Concentration (µM)
Concentration (dilutions)
mCherry
CTG
Mock
b
c
d
e
mCherry
CTG
Virus
Virus - Mock
Synergy distribution
IFNa2a (ng/mL)
EIDD-2801 (µM)
Cycloheximide (µM)
Conv. Serum (dilution factor)
Camostat (µM)
Infected cells (%)
Viable cells (%)
ZIP synergy score
Most synergistic area

Figure S5. Synergistic IFNa2a-based combinations against SARS-CoV-2-mCherry infection in Calu-3 cells. (**a**) Calu-3 cells were treated with increasing concentrations of a drug and infected with the SARS-CoV-2-mCherry or mock. After 48 h, the virus-mediated mCherry expression was measured (red curves). After 72 h, viability of virus- and mock-infected cells was determined using a CTG assay (yellow and blue curves, respectively). Mean ± SD; $n = 3$. (**b-e**, left panels) The 6 × 6 dose–response matrices and interaction landscapes of IFNa2a and remdesivir obtained using fluorescence analysis of SARS-CoV-2-mCherry-infected Calu-3 cells. ZIP synergy score was calculated for the drug combinations. (**b-e**, right panels) The 6 × 6 dose–response matrices and interaction landscapes of IFNa2a and remdesivir obtained using a cell viability assay (CTG) on mock-, and SARS-CoV-2-mCherry-infected Calu-3 cells. The selectivity for the indicated drug concentrations was calculated (selectivity = efficacy-(100-Toxicity)). ZIP synergy scores were calculated for indicated drug combinations.

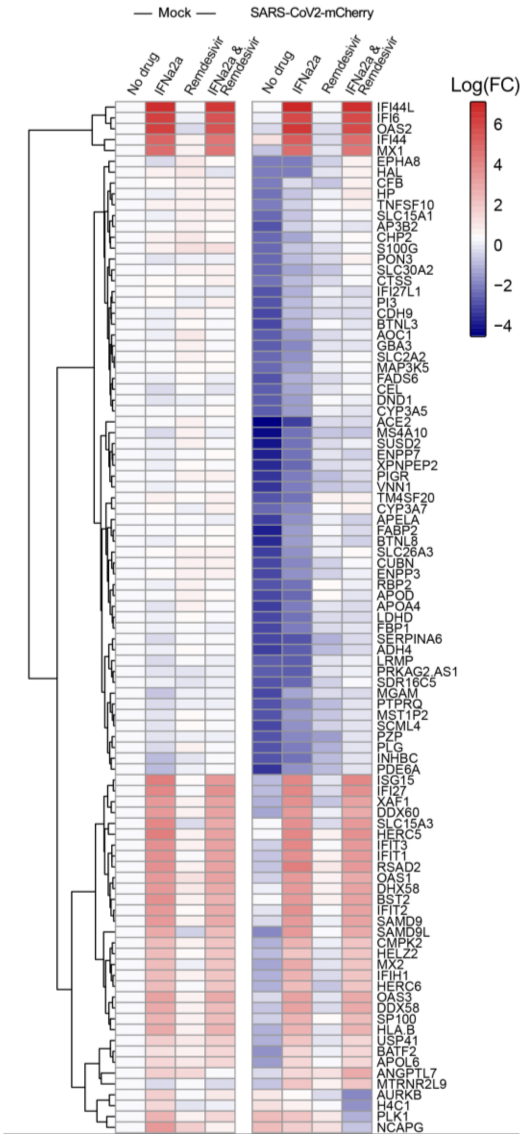

Figure S6. Effect of IFNa2a-remdesivir on transcription of host genes in human lung organoids (LOs). LOs were treated with 0,5 μM remdesivir, 5 ng/mL IFNa2a, a combination thereof, or vehicle; then infected with SARS-CoV-2-mCherry (moi = 0,1) or mock. After 48 h, total RNA was extracted and sequenced. A heatmap of the most variable cellular genes affected by treatment and virus infection is shown. Each cell is colored

according to the log2-transformed expression values of the samples, expressed as fold-change relative to the nontreated mock-infected control. Cut-off - 2.

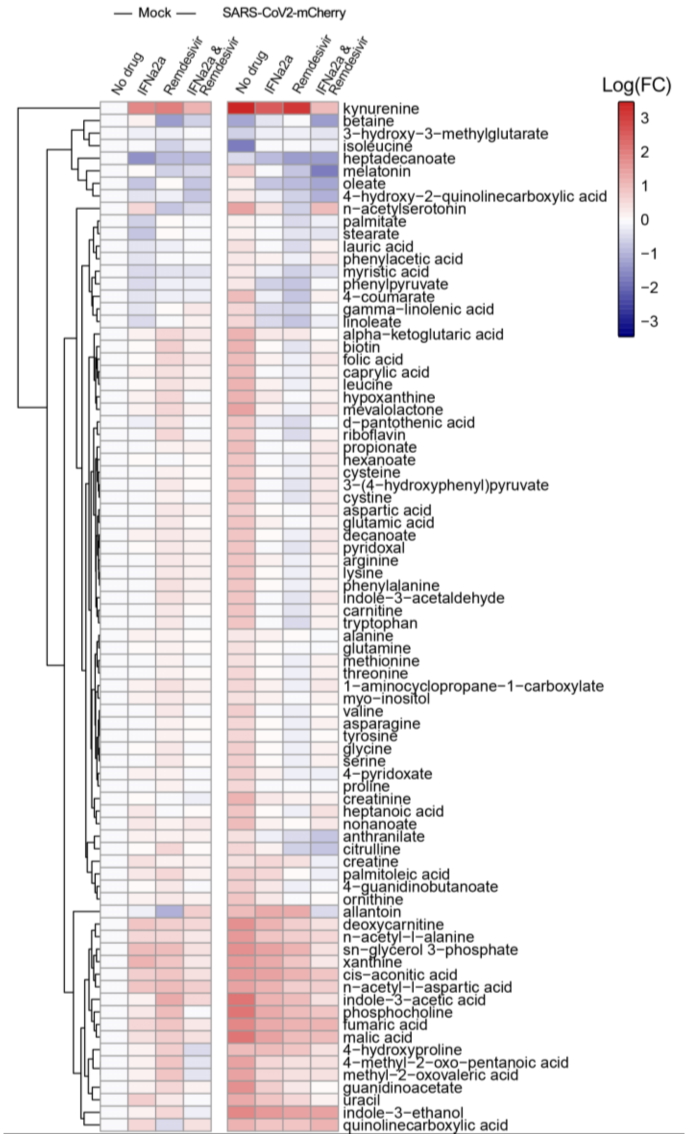

Figure S7. Effect of IFNa2a-remdesivir on metabolism in human lung organoids (LOs). LOs were treated with 0,5 µM remdesivir, 5 ng/mL IFNa2a, a combination thereof, or vehicle; then infected with SARS-CoV-2-mCherry (moi = 0,1) or mock. After 48 h, the cell culture supernatants were collected, and metabolite levels were determined by LC-MS/MS. A heatmap of the most affected metabolites is shown. Each cell is colored according to the log2-transferred profiling values of samples, expressed as fold-change relative to the mock control.

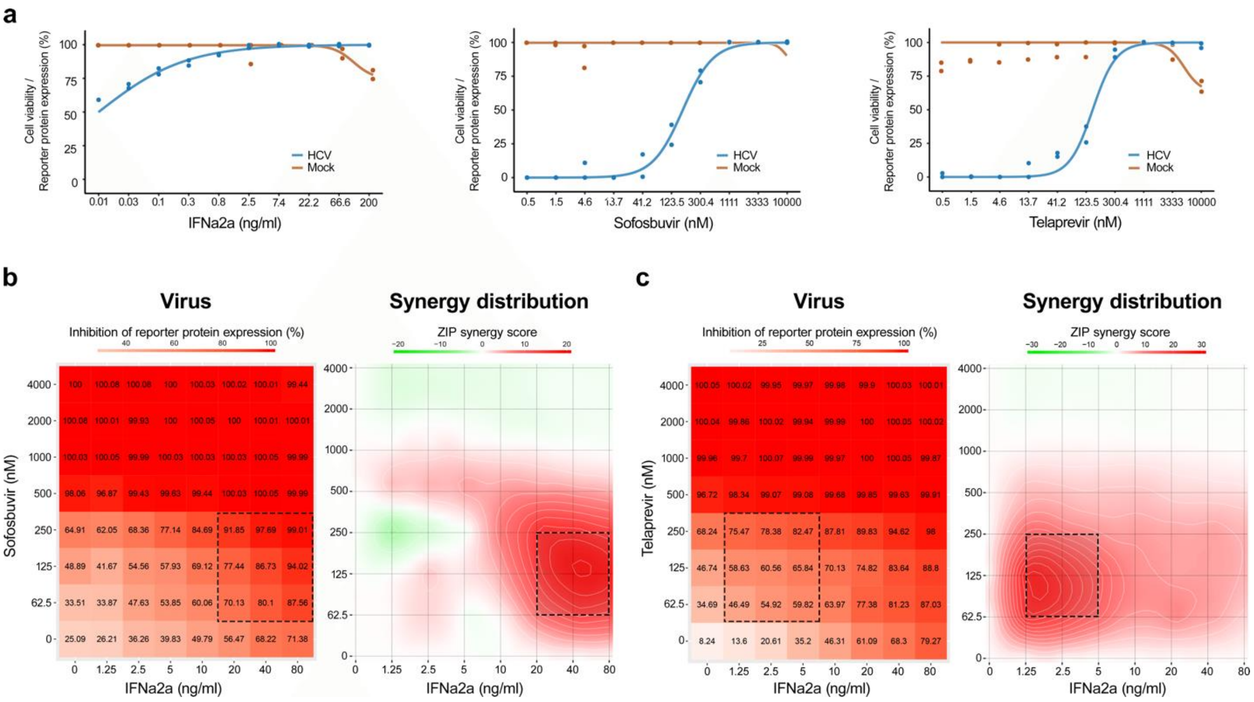

Figure S8. Combinations of IFNa2a with sofosbuvir or telaprevir reduce HCV-mediated GFP expression in Huh-7.5 cells. (**a**) Huh-7.5 cells were treated with increasing concentrations of IFNa2a, sofosbuvir, or telaprevir and infected with HCV. After 72 h, the HCV-mediated GFP expression was measured (blue curves). The total number of cells was used as marker for cytotoxicity (red curves). Mean ± SD; *n* = 3. (**b**,**c**) The interaction landscape of IFNa2a-sofosbuvir and IFNa2a-telaprevir, measured using HCV-mediated GFP expression in Huh-7.5 cells (left panels). The interaction landscapes of drug combinations (right panels).

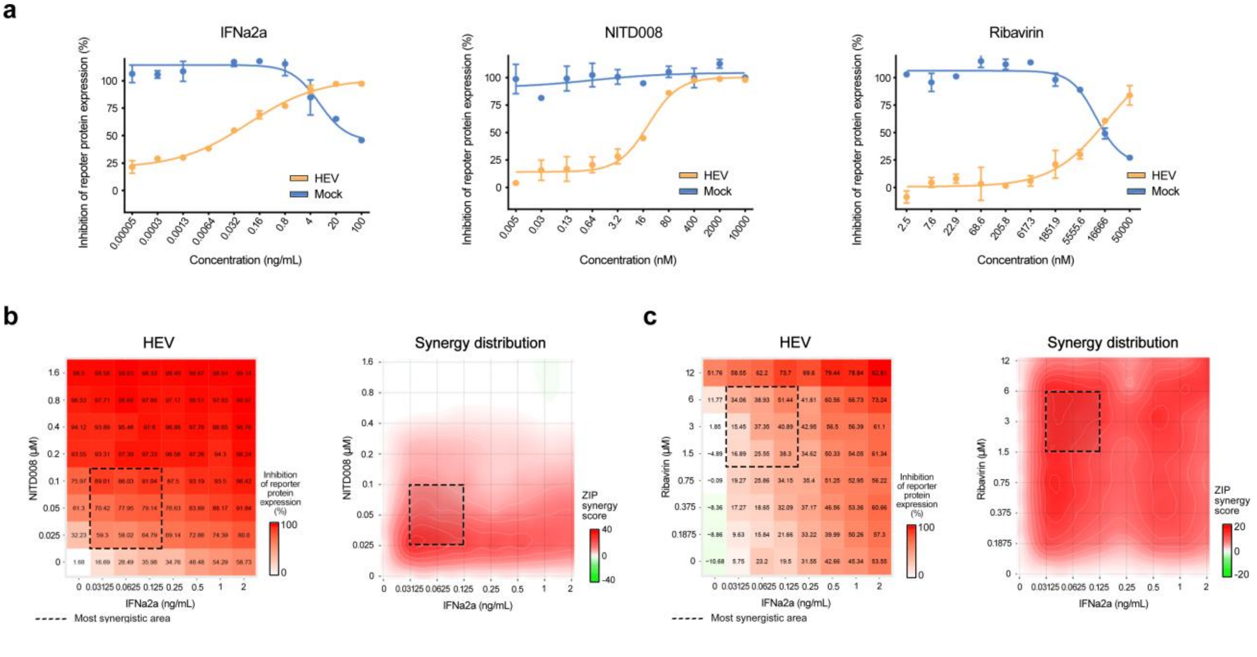

Figure S9. Combinations of IFNa2a with ribavirin or NITD008 reduce HEV-mediated GFP expression in Huh-7.5 cells. (**a**) Huh-7.5 cells were transfected with p6-GFP sub-genomic HEV RNA or mock. After 24 h cells were treated with increasing concentrations of IFNa2a, NITD008 or ribavirin. After 72 h, the HEV-

mediated GFP expression was measured (blue curves). Total number of cells was used as marker for cytotoxicity (red curves). Mean ± SD; n = 3. (**b**,**c**) The interaction landscape of IFNa2a-ribavirin and IFNa2a-NITDD008 were measured using HEV-mediated GFP expression in Huh-7.5 cells (left panels). The interaction landscapes of drug combinations (right panels).

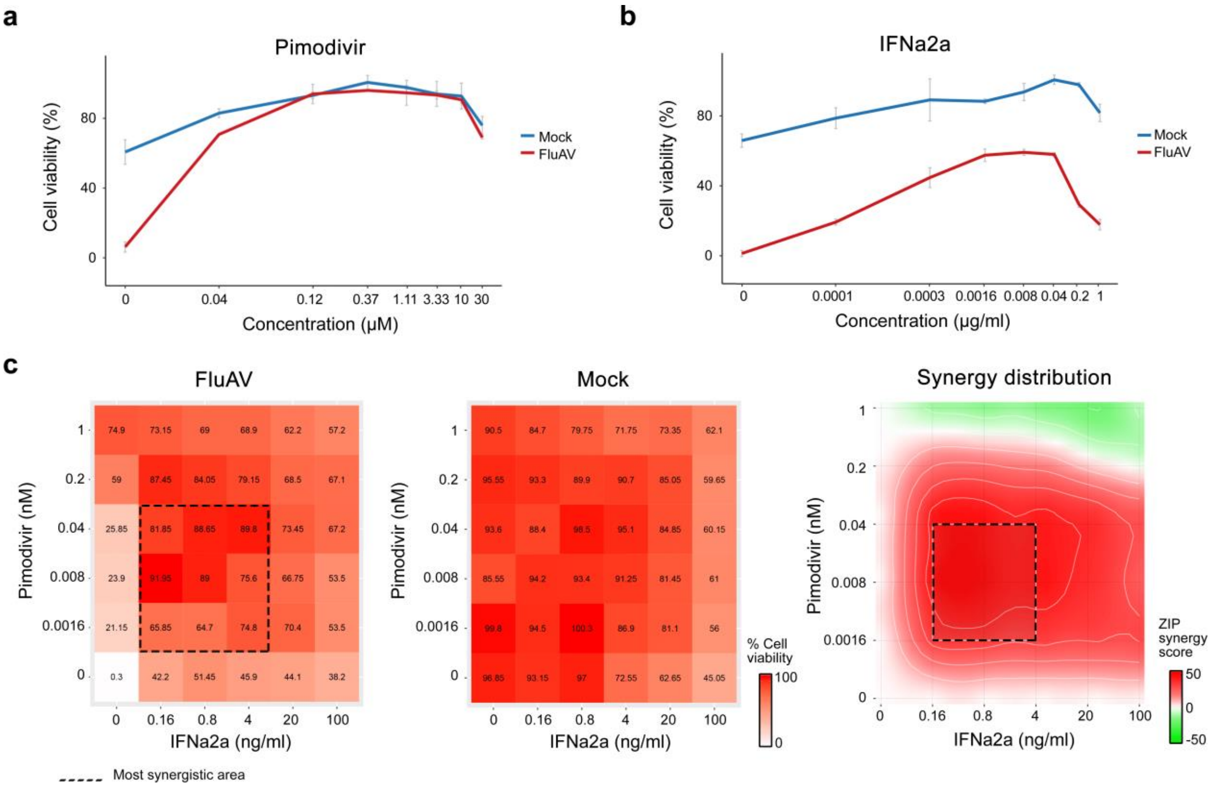

Figure S10. Combination of pimodivir-IFNa2a reduces FluAV infection in A549 cells. (**a**,**b**) A549 cells were treated with increasing concentrations of pimodivir or IFNa2a and infected with the FluAV (moi = 0.5) or mock. After 48 h, cell viability was determined using a CTG assay. Mean ± SD; n = 3. (**c**) The interaction landscape of IFNa2a and pimodivir in FluAV- and mock infected A549 cells measured using CTG (left panels). The interaction landscape of both drugs showing synergy of the drug combination (right panel).

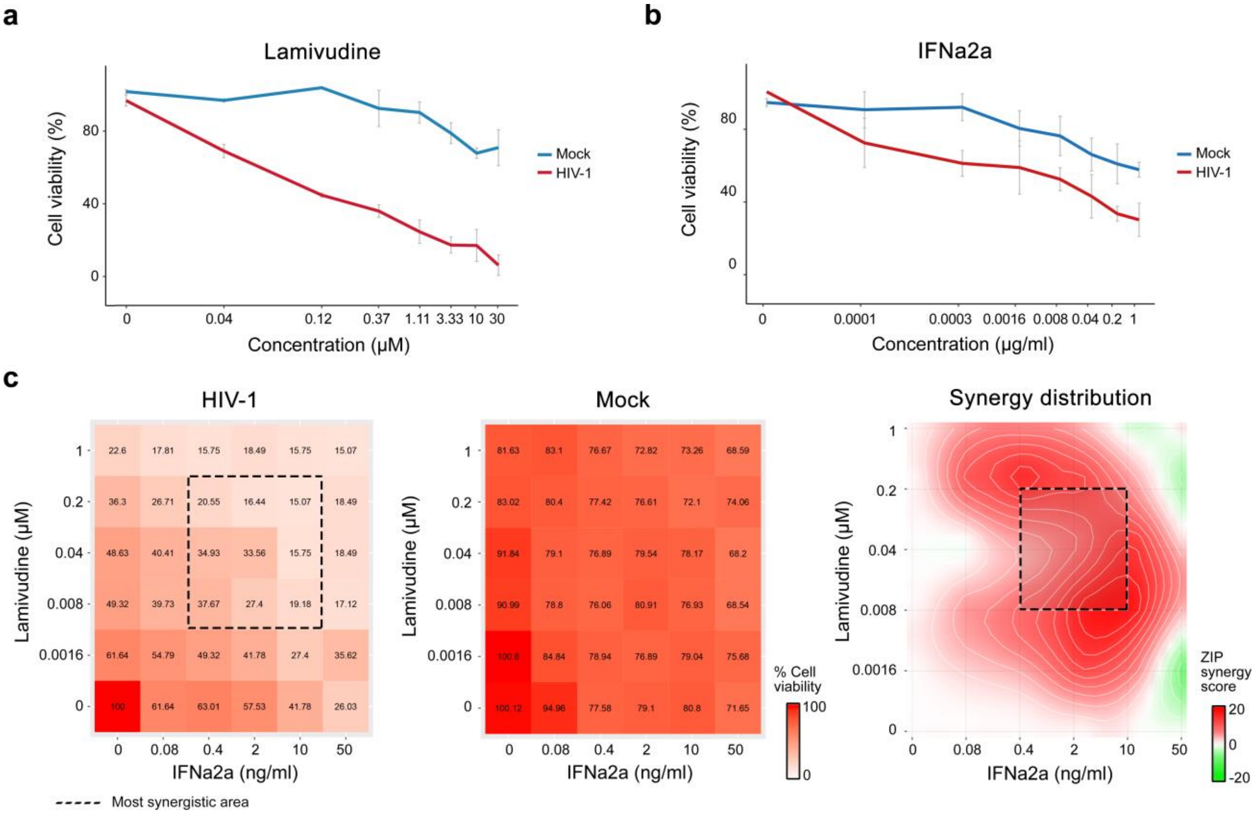

Figure S11. Combination of lamivudine-IFNa2a reduces HIV-1 virus infections in TZM-bl cells. (**a**,**b**) TZM-bl cells were treated with increasing concentrations of lamivudine or IFNa2a and infected with the HIV-1 or mock. After 48 h, the HIV-mediated luciferase expression was measured. Viability of mock-infected cells was determined using the CTG assay. Mean ± SD; n = 3. (**c**) The interaction landscape of IFNa2a and lamivudine in HIV-1- and mock-infected TZM-bl cells measured using HIV-1-mediated luciferase expression and CTG assay, respectively (left panels). The interaction landscape of both drugs showing synergy of their combination (right panel).
